# Increased Risk for Adhesive Capsulitis of the Shoulder following Cervical Disc Surgery

**DOI:** 10.1038/srep26898

**Published:** 2016-05-27

**Authors:** Jiunn-Horng Kang, Herng-Ching Lin, Ming-Chieh Tsai, Shiu-Dong Chung

**Affiliations:** 1 Department of Physical Medicine and Rehabilitation, Taipei Medical University Hospital, Taipei, Taiwan; 2 Department of Physical Medicine and Rehabilitation, School of Medicine, College of Medicine, Taipei Medical University, Taiwan; 3 Sleep Research Center, Taipei Medical University Hospital, Taipei, Taiwan; 4 Department of Internal Medicine, Cathay General Hospital, Hsinchu Branch, Taiwan; 5 Department of Surgery, Far Eastern Memorial Hospital, Banciao, Taiwan; 6 Graduate Program in Biomedical Informatics, College of Informatics, Yuan-Ze University, Chung-Li, Taiwan

## Abstract

Shoulder problems are common in patients with a cervical herniated intervertebral disc (HIVD). This study aimed to explore the incidence and risk of shoulder capsulitis/tendonitis following cervical HIVD surgery. We used data from the Taiwan “Longitudinal Health Insurance Database”. We identified all patients who were hospitalized with a diagnosis of displacement of a cervical HIVD and who underwent cervical surgery (*n* = 1625). We selected 8125 patients who received cervical HIVD conservative therapy only as the comparison group matched with study patients. We individually tracked these sampled patients for 6 months to identify all patients who received a diagnosis of shoulder tendonitis/capsulitis. We found that incidence rates of shoulder tendonitis/capsulitis during the 6-month follow-up period were 3.69 (95% CI: 2.49~5.27) per 100 person-years for the study group and 2.33 (95% CI: 1.89~2.86) per 100 person-years for the comparison group. Cox proportional hazard regressions showed that the adjusted hazard ratio for shoulder tendonitis/capsulitis among patients who underwent cervical disc surgery was 1.66 (95% CI = 1.09~2.53) when compared to comparison group. We concluded that patients who underwent surgery for a cervical HIVD had a significantly higher risk of developing shoulder capsulitis/tendonitis in 6 months follow-up compared to patients who received cervical HIVD conservative therapy only.

A cervical herniated intervertebral disc (HIVD) is a common disease that manifests as neck pain and upper limb pain. Although most symptomatic cervical HIVDs are self-limited and can successfully be treated with conservative treatments^1^, surgery is still indicated in HIVD patients with refractory pain, persistent pain, or significant neurological consequences^2^,^3^. Common surgical management options for cervical HIVD are an anterior cervical discectomy and fusion (ACDF), a posterior foraminotomy, and total disc replacement.4 Surgery for a cervical HIVD usually provides a high success rate of relieving radicular pain and carries a low risk for complications by an experienced neurosurgeon. Some complications were reported following cervical HIVD surgery, such as pseduoarthrosis, adjacent level degeneration, recurrent laryngeal or hypoglossal nerve injury, and Honer’s syndrome^4^,^5^.

The neck and shoulders are highly anatomically and functionally associated. Several muscles and ligaments link the motion of the neck and shoulders such as the trapezius, levator scapularis, rhomboid major, etc. In addition, neurological control of the shoulder girdle muscles is mainly supplied from cervical roots particularly from C5/C6 roots. Therefore, it is not uncommon that a patient has coincident cervical and shoulder problems. Some patients with a cervical HIVD may suffer from a “neurogenic” frozen shoulder. Although the data are scanty, shoulder periarthritis, also known as frozen shoulder, was reported in about 10% of cervical HIVD patients in a case series study. Interestingly, that study reported the prevalence of shoulder capsulitis increased to 22% postoperatively after patients underwent cervical surgery for an HIVD^6^.

Although the strong association between neck and shoulder problems has long been recognized, the specific prevalence and risk of shoulder capsulitis/tendonitis following surgery for cervical HIVD are still not well known. In the present study, we explored the incidence and risk of developing shoulder capsulitis and tendonitis following surgery for a cervical HIVD in a large population-based study. The data are critical for developing adequate clinical management and providing a fundamental basis for postoperative management.

## Methods

### Database

This study used data sourced from the “Longitudinal Health Insurance Database 2005 (LHID2005)” released by the Taiwan National Health Research Institute (NHRI) in 2006. Taiwan began a single-payer National Health Insurance (NHI) program in 1995 to provide affordable health care for all of the island’s residents. The LHID2005 contains all medical claims data for 1,000,000 beneficiaries, randomly sampled from all enrollees (*n* = 25.68 million) covered under the NHI program. The Taiwan NHRI reports that there were no statistically significant differences in the distributions of age, gender, or healthcare costs between patients in the LHID2005 and all enrollees. Therefore, the LHID2005 provides a unique opportunity to examine the risk of shoulder capsulitis/tendonitis in patients who had undergone surgery for a cervical HIVD.

### Study Sample

This study consisted of a study group and a comparison group. We identified all patients who were hospitalized with a diagnosis of displacement of a cervical intervertebral disc (ICD-9-CM code 722.0) and underwent cervical fusion, anterior technique (ICD-9-CM procedure code 810.2) or excision of the intervertebral disc (ICD-9-CM procedure code 805.1) between January 1, 2001 and December 31, 2013 (*n* = 1661). We defined the date of undergoing cervical disc surgery as the index date for the study group. We then excluded patients who were less than 18 years old in order to limit our study sample to the adult population (*n* = 1). Furthermore, in order to increase the likelihood of identifying only new shoulder tendonitis/capsulitis cases, we excluded 35 patients who had ever received a diagnosis of shoulder tendonitis/capsulitis (ICD-9-CM codes 726.0, 726.1, or 726.2) within 6 months prior to the index date. Finally, 1625 patients were included in the study group.

As for comparison group, we first identified 17,391 patients who received a first-time diagnosis of displacement of a cervical intervertebral disc (ICD-9-CM code 722.0) in ambulatory care centers or hospitalizations between 1 January 2001 and 31 December 2013. We designated the date of receiving their first-time diagnosis of cervical HIVD as index date for comparison group. We then excluded those patients who had ever undergone cervical disc surgery after the index date. Thereafter, using the SAS proc surveyselect program, we randomly extracted 8,125 comparison patients (five for every patient who underwent a cervical disc surgery) from the remaining patients matched with the study patients in terms of gender, age group (18~40, 40~49, 50~59, 60~69, and >69 years), and index year. We also assured that none of the selected comparison patients had ever received a diagnosis of shoulder tendonitis/capsulitis within 6 months prior to the index date. Ultimately, we individually tracked these sampled patients (*n* = 9750) for 6 months from their index date to identify all patients who subsequently received a diagnosis of shoulder tendonitis/capsulitis during the follow-up period.

### Statistical Analysis

We used the SAS statistical package (SAS System for Windows, vers. 8.2) to perform all statistical analyses. Pearson χ^2^ tests and *t*-tests were used to explore differences in sociodemographic characteristics (age, sex, monthly insured income amount, and geographic region and level of urbanization of the community in which the patient resided) between the study and comparison groups. Cox proportional hazard regressions, which do not invoke proportionality assumptions, were used to calculate the hazard of shoulder tendonitis/capsulitis between these two groups. A two-sided *p* value of ≤0.05 was considered significant in this study.

## Results

Table 1 shows the distribution of sociodemographic characteristics between patients who underwent cervical disc surgery and the comparison group. There was no significant difference in the mean age between these two groups (*p* = 0.165). Pearson χ^2^ tests revealed that patients who underwent cervical disc surgery were more likely to reside in the northern part of Taiwan (*p* < 0.001) than patients in the comparison group.

Table 2 shows the distribution of shoulder tendonitis/capsulitis during the 6-month follow-up period between patients who underwent cervical disc surgery and comparison patients. We found that of the total sample of 9750 patients, 125 patients (1.28%) subsequently received a diagnosis of shoulder tendonitis/capsulitis during the 6-month follow-up period, 30 (1.85% of the patients who underwent a cervical disc surgery) from the study group and 95 (1.17% of patients in the comparison group) from the comparison group. Incidence rates of shoulder tendonitis/capsulitis during the 6-month follow-up period were 3.69 (95% CI: 2.49~5.27) per 100 person-years for the study group and 2.33 (95% CI: 1.89~2.86) per 100 person-years for the comparison group. The log-rank test indicated that there was a significant difference in shoulder tendonitis/capsulitis-free survival rates between patients who underwent cervical disc surgery and comparison patients (*p* = 0.002).

Table 3 shows the crude and adjusted hazard ratios (HRs) of shoulder tendonitis/capsulitis within the 6-month follow-up period comparing patients who underwent cervical disc surgery and comparison patients. The crude HR for shoulder tendonitis/capsulitis for patients who underwent cervical disc surgery was 1.59 (95% CI = 1.05~2.41) compared to comparison patients. Furthermore, the HR for shoulder tendonitis/capsulitis for patients who underwent cervical disc surgery was 1.66 (95% CI = 1.09~2.53) times higher over the 6-month follow-up period, after adjusting for monthly income, level of urbanization of the community and geographic region in which the patient resided.

We have further analyzed the risk of tendonitis/capsulitis during the 1-year follow-up period. We found that the HR for shoulder tendonitis/capsulitis for patients who underwent cervical disc surgery was 1.56 (95% CI = 1.10~2.21) times higher over the 1-year follow-up period. The relationship between cervical disc surgery and shoulder tendonitis/capsulitis still sustained even during the 1-year follow-up period.

## Discussion

We found an increased risk for shoulder capsulitis and tendonitis in the population with a cervical HIVD following cervical surgery in a large population-based cohort study. The adjusted HR for developing shoulder capsulitis/tendonitis following cervical surgery was 1.66 compared to patients who received cervical HIVD conservative therapy only. Although the overall prevalence of shoulder capsulitis/tendonitis was relatively minor (1.85% in patients who received cervical HIVD surgery), there are significant numbers of patients receiving cervical surgery for HIVD annually. Therefore, recognition of this issue can improve patient management and satisfaction following cervical surgery for an HIVD.

Even though the concept regarding an association between cervical and shoulder disorders has been noted for a long time, epidemiological data are still very limited. A study showed more evidence of degenerative changes at C5/6 and C6/7 intervertebral discs in patients with shoulder capsulitis/tendonitis than a control population^6^. However, that cross-sectional study was limited in being unable to establish a temporal association between the two conditions. Another observational case series study noted an increased prevalence of shoulder periarthritis/capsulitis following cervical surgery for an HIVD^6^. However, the overall risk of shoulder capsulitis/tendonitis following cervical surgery could not be estimated in this study since it lacked a control population. To our best knowledge, the present investigation is the first study to explore the prevalence and risk of developing shoulder capsulitis/tendonitis following surgery for a cervical HIVD with a large population cohort. Our study can add knowledge of this issue and should be useful in providing adequate patient information and facilitate postoperative care in this population.

The mechanisms of a “paradoxically” increased risk of shoulder capsulitis/tendonitis following cervical surgery may be complex. To our best knowledge, there is very scanty data to indicate and explore the pathomechanism of the association between cervical HIVD surgery and postoperative shoulder capsulitis. Therefore, we can only raise several hypotheses in present study based on current available works and observations. First, relative immobilization and reduced movements of the neck and shoulder region following surgery may increase the risk of developing shoulder capsulitis^7^. In addition, postoperative pain may further exacerbate this condition. Second, few patients may have arm palsy or weakness following cervical decompression surgery for an HIVD which is known as C5 palsy syndrome^8^,^9^. The cause of C5 palsy syndrome is still not well understood, and multiple factors were proposed to be associated with C5 palsy syndrome, such as direct C5 root injury, root tethering, spinal cord ischemia, reperfusion injury, and segmental spinal cord disease^9^. Patients with C5 palsy syndrome present remarkable weakness involving muscles innervated by the C5 root. Although most patients with C5 palsy syndrome gradually recover postoperatively, temporary shoulder girdle weakness and dysfunction may put patients at high risk of developing postoperative shoulder capsulitis/tendonitis. Third, successful cervical decompression surgery may lead to significant pain relief which may allow patients to increase the functional use of their upper extremities in their daily activities following surgery. The increased daily upper extremity use may contribute to a risk of overuse and injury of the shoulder. Nevertheless, further study is still need to verify these hypotheses.

Shoulder capsulitis/tendonitis is a common musculoskeletal problem in the general population. The shoulder is the most mobile joint of the body and is critical to performing upper extremity functions. Although most cases of shoulder capsulitis/tendonitis can successfully be treated, shoulder capsulitis/tendonitis can still be associated with pain and disability for a considerable period and an increased medical burden^10^. The increased risk of shoulder capsulitis/tendonitis may be preventable following cervical surgery for a cervical HIVD. Several steps should be considered. Careful preoperative evaluation of shoulder mobility and the functional status is helpful in differentiating some patients with comorbid shoulder capsulitis/tendonitis. Intensive treatment of comorbid shoulder problems prior to surgery can optimize postoperative outcomes and improve patient satisfaction. Patients who undergo cervical surgery should be instructed about an adequate mobilization program of the shoulders. A strengthening program of the shoulder girdle should be emphasized for patients with C5/6 radiculopathy and/or shoulder muscle weakness.

Aggressive pain control following surgery is fundamental. Inadequate pain control can promote immobilization and further loss of function. Careful monitoring of the shoulder’s range of motion and function postoperatively is necessary. Early detection and aggressive management of shoulder capsulitis/tendonitis are linked to better outcomes and prevention of functional loss. Neurological recovery in patients with cervical radiculopathy could be timely and incomplete even after successful surgical decompression of the cervical roots. Prolonged cervical radiculopathy may cause weakness and dysfunction of the shoulder girdle which can promote shoulder capsulitis. For patients with severe radiculopathy preoperatively or C5 palsy syndrome postoperatively, preventive rehabilitation may be needed to minimize immobilization and inactivity of the shoulder.

Limitations of present study should be addressed. First, shoulder capsulitis/tendonitis may have been mis-coded or uncoded in the registry-based database, particularly of patients who had minor symptoms and did not seek medical services. In addition, some shoulder symptoms may mimic radicular pain which may have been undiagnosed preoperatively in patients with comorbid shoulder capsulitis/tendonitis. Second, the neurological status and level of patients who underwent cervical HIVD surgery could not be determined from the database. In theory, patients with more-severe cervical radiculopathy involving C5/6 may have a higher risk of developing shoulder problems. Third, some variables such as occupation, trauma history, family history, physical activity, cigarette smoking, and obesity which may have confounded our findings could not be determined in the present study. Fourth, we did not analyze the effects by the surgery types for cervical HIVD. Complications of cervical spine surgery are usually dependent on the type of procedure. The risk for developing shoulder capsulitis/tendonitis may vary with different types of surgery. Finally, the causes of cervical HIVD could not be determined in our database. Degenerative and traumatic HIVDs may be associated with different risks of developing shoulder capsulitis/tendonitis.

## Conclusions

Patients who underwent surgery for a cervical HIVD had a higher risk of subsequently developing shoulder capsulitis/tendonitis postoperatively compared to patients who received cervical HIVD conservative therapy only in 6 months follow-up in a large population-based cohort study. Adequate monitoring and management of shoulder problems in patients with a cervical HIVD surgery are suggested.

## Additional Information

**How to cite this article**: Kang, J.-H. *et al*. Increased Risk for Adhesive Capsulitis of the Shoulder following Cervical Disc Surgery. *Sci. Rep*. **6**, 26898; doi: 10.1038/srep26898 (2016).

## Figures and Tables

**Table 1:** Comparison of patients who underwent cervical disc surgery and a comparison group in relation to sociodemographic characteristics in Taiwan (N = 9750).

Variables	Patients who underwent cervical disc surgery *N* = 1625		Comparison patients *N* = 8125		*p* value
	No.	%	No.	%	
Age, mean (standard deviation) (years)	54.4 (13.1)		53.9 (13.9)		0.165
Male	939	57.8	4695	57.8	>0.999
Monthly insured income					0.001
≤NT$15,840	591	36.4	2965	36.5	
NT$15,841~25,000	605	37.2	2685	33.1	
≥NT$25,001	429	26.4	2475	30.5	
Urbanization level					<0.001
1 (highest level)	495	30.5	2200	27.1	
2	463	28.5	2605	32.1	
3	238	14.6	1325	16.3	
4	238	14.6	1265	15.5	
5 (lowest level)	191	11.8	730	9.0	
Geographic region					<0.001
Northern	830	51.2	3590	44.2	
Central	423	26.0	2750	33.9	
Southern	345	21.2	1620	19.9	
Eastern	27	1.6	165	2.0	

The average exchange rate in 2013 was US$1 ≈ New Taiwan (NT)$31.

**Table 2:** Relationships of cervical disc surgery with shoulder tendonitis/capsulitis during the 6-month follow-up period for patients who underwent cervical disc surgery and comparison patients (n = 9750).

Presence of shoulder tendonitis/capsulitis	Total sample		Patients who underwent cervical disc surgery		Comparison patients	
	No.	%	No.	%	No.	%
Six-month follow-up period						
Yes	125	1.28	30	1.85	95	1.17
Incidence rate per 100 person-years (95% CI)	2.56 (2.13~3.06)		3.69 (2.49~5.27)		2.33 (1.89~2.86)	

CI, confidence interval.

**Table 3:** Crude and adjusted hazard ratios (HRs) for shoulder tendonitis/capsulitis.

Variable	Shoulder tendonitis/capsulitis occurrence	
	Crude HR (95% CI)	Adjusted HR (95% CI)
Cervical disc surgery	1.59* (1.05~2.41)	1.66* (1.09~2.53)
Monthly insured income		
≤NT$15,840	1.00	1.00
NT$15,841~25,000	0.72 (0.46~1.11)	0.74 (0.47~1.16)
≥NT$25,001	0.96 (0.63~1.46)	0.98 (0.64~1.50)
Urbanization level		
1 (highest level)	1.00	1.00
2	5.71*** (2.57~12.69)	6.87*** (3.08~15.34)
3	8.54*** (3.78~19.30)	11.07*** (4.82~25.42)
4	6.49*** (2.80~15.05)	10.29*** (4.29~24.68)
5 (lowest level)	5.93*** (2.38~14.72)	9.17*** (3.55–23.64)
Geographic region		
Northern	1.00	1.00
Central	0.67 (0.45~1.02)	0.42*** (0.27~0.66)
Southern	0.25*** (0.12~0.53)	0.19*** (0.09~0.40)
Eastern	4.51*** (2.45~8.31)	2.55** (1.35~4.83)

*Note*: *p < *0.05*; **p < *0.01*; ***p < *0.001*; adjusted HRs were all derived from a Cox regression model and were adjusted for all other variables; CI, confidence interval; The average exchange rate in 2013 was US$1 ≈ New Taiwan (NT)$31.
